# Prenatal Transfer of Gut Bacteria in Rock Pigeon

**DOI:** 10.3390/microorganisms8010061

**Published:** 2019-12-30

**Authors:** Maurine W. Dietz, Joana F. Salles, Bin-Yan Hsu, Cor Dijkstra, Ton G. G. Groothuis, Marco van der Velde, Yvonne I. Verkuil, B. Irene Tieleman

**Affiliations:** 1 Groningen Institute for Evolutionary Life Sciences, University of Groningen, Nijenborgh 7, 9747AG Groningen, The Netherlands; 2 Department of Biology, University of Turku, 20014 Turku, Finland

**Keywords:** gut microbiome, oviparous animals, prenatal transmission, rock pigeon

## Abstract

Vertebrates evolved in concert with bacteria and have developed essential mutualistic relationships. Gut bacteria are vital for the postnatal development of most organs and the immune and metabolic systems and may likewise play a role during prenatal development. Prenatal transfer of gut bacteria is shown in four mammalian species, including humans. For the 92% of the vertebrates that are oviparous, prenatal transfer is debated, but it has been demonstrated in domestic chicken. We hypothesize that also non-domestic birds can prenatally transmit gut bacteria. We investigated this in medium-sized Rock pigeon (*Columba livia*), ensuring neonates producing fair-sized first faeces. The first faeces of 21 neonate rock pigeons hatched in an incubator, contained a microbiome (bacterial community) the composition of which resembled the cloacal microbiome of females sampled from the same population (*N* = 5) as indicated by multiple shared phyla, orders, families, and genera. Neonates and females shared 16.1% of the total number of OTUs present (2881), and neonates shared 45.5% of their core microbiome with females. In contrast, the five females shared only 0.3% of the 1030 female OTUs present. These findings suggest that prenatal gut bacterial transfer may occur in birds. Our results support the hypothesis that gut bacteria may be important for prenatal development and present a heritability pathway of gut bacteria in vertebrates.

## 1. Introduction

Gut microbes (i.e., bacteria, fungi, viruses, protists) have complex symbiotic relationships with their hosts. These relationships can be mutualistic: gut bacteria exert profound beneficial effects on host nutrition and metabolism and play a vital role in immune functioning, while they benefit from a relative constant habitat with a continuous food flow provided by the host [1,2,3,4,5]. Gut bacteria are essential for proper postnatal development [1,2,3,6,7,8], and the initial gut microbiome has life-long effects on the immunity, metabolism, and health of the host [1,9,10,11]. There are indications that gut bacteria modulate fetal immune and metabolic systems [2,8,12], e.g., exposure to microbial antigens may explain the early onset of fetal immunity [13]. Gut bacteria may thus also be important for prenatal development. For gut bacteria, prenatal transfer ensures a direct route to new hosts, which may be especially significant for bacteria that are adapted to living in the gut and survive poorly outside a host. Hence, host-bacteria co-evolution may have selected for gut bacteria capable of prenatal transfer and hosts controlling this prenatal process.

Indeed, prenatal mother-to-offspring transfer of gut bacteria is widespread among animals [1,2,8,10,14]. Occurrence of prenatal transfer of live gut bacteria in vertebrates was shown via an experiment in mice by Jiménez and co-authors [15]. They orally inoculated pregnant mice with a genetically labelled *Enterococcus fecium* strain and found this labelled bacterium in the aseptically collected meconium of full-term fetuses delivered via caesarean section. The meconium of full term fetuses of control mothers that were not inoculated, did not contain the labelled *E. fecium*. To date, prenatal transfer of gut bacteria has been demonstrated in four mammalian species: mice (*Mus musculus*) [15], rats (*Rattus norvegicus*) [16], Japanese macaques (*Macaca fuscata*) [17], and repeatedly in humans [2,8]. Presumably prenatal gut bacteria transfer occurs continuously throughout gestation in mammals [2,8,10]. Note that prenatal transfer of gut bacteria is not always found, e.g., Leblois et al. [18] did find intestinal bacteria in the umbilical cord of Landrace piglets (*Sus scrofa domesticus*), but meconium bacterial DNA concentrations were below detection limits.

We hypothesize that if gut bacteria are important for pre- and postnatal development, prenatal transfer should also occur in oviparous vertebrate species who make up ~92% of the vertebrates, even though embryonic development takes place outside the mother and continuous transfer is unlikely. In two Arctic-breeding shorebird species embryonic guts contained bacterial abundances similar to negative controls [19], indicating absence or very low bacterial load. However, two other studies on embryonic guts in birds, in domestic chicken (*Gallus gallus domesticus*), found that chicken embryos had well-established gut bacterial communities [20,21]. Broadening the range of bird taxa and habitats in which embryonic/neonate gut bacteria are studied is needed, and therefore, we investigated gut bacteria in semi-wild rock pigeons (*Columba livia*), a medium-sized bird. Our approach was to collect the first feces after eggs were hatched in an adult-free environment, a sampling approach that would be viable in many other oviparous taxa including free-living animals. We compared the bacterial communities (henceforth called microbiome) of chicks with cloacal microbiomes of females randomly chosen from the same population. In addition, we reanalyzed published data on chicken and mammal neonates to compare them with our results. If prenatal transfer of gut bacteria does occur in birds, we expect to find a well-established avian microbiome in the neonate rock pigeons’ first feces that is comparable to the adult female cloacal microbiome. Furthermore we expect a lower similarity between neonatal and female microbiomes in birds than in mammals, because in birds prenatal transfer presumably occurs mainly prior to oviposition, whereas in mammals transfer may occur throughout gestation [2,8,10].

## 2. Materials and Methods

### 2.1. Ethics Statement

The experiment and animal care procedures were approved by the Animal Welfare Committee of the University of Groningen (DEC no. 5635F) and complied with the Dutch law.

### 2.2. Animals

We used captive rock pigeons from our outbred wild-caught colony housed at the Groningen Institute for Evolutionary Life Sciences and housed a total of 48 pairs each in separate outdoor aviaries (4.01 m × 1.67 m × 2.2 m, l × w × h). The rock pigeons received food (seed mixture for *Streptopelia* species, KASPER^TM^ 6721, and standard food for *Columba* species, KASPER^TM^ 6712, Kasper Faunafood, Woerden, Netherlands), grit and water ad libitum. The birds took part in a cross-incubation and cross-fostering experiment on androgen-mediated effects in relation to the environment, conducted between April and June 2013 [22,23]. Briefly, for the androgen experiment, we injected first-laid eggs of the two-egg-clutches of experimental and colony pairs within 3 days post laying, thus before incubation started, with 50 μL solution of crystalline testosterone (Sigma-Aldrich, VETRANAL™, Darmstadt, Germany) dissolved in sterilized sesame oil in the egg yolk, resulting in testosterone neonates, or 50 μL pure sesame oil, resulting in control neonates. To minimize contamination, we sterilized the injection location with 70–96% ethanol prior to injection, used sterile needles/syringes, and sealed the hole with a small piece (~25 mm^2^) of artificial skin (Hansaplast^TM^, Beiersdorf AG, Hamburg, Germany). After injection, the eggs were randomly assigned to the breeding pairs, forming two-egg clutches consisting of a control and testosterone egg. The second eggs of the clutches were returned to non-experimental breeding pairs in the colony.

The injection procedure is a potential source of contamination. However, the results indicate that our precautions sufficiently avoided contamination. Firstly, hatchability (51–55% for testosterone eggs and 46–51% for control eggs [22]) was close to that of wild feral pigeons (*Columba livia*, 68.5% [24]), indicating that the injection procedure did not lead to contamination of the egg, as contamination with foreign bacteria is expected to lead to a considerable decrease in hatchability. Secondly, if contamination of the egg had taken place, it is unlikely that the invasive foreign bacteria would be able to reach the embryonic gut, given the differences in the environmental constraints driving bacterial fitness in- and outside the host. Lastly, if the potential contamination is able to establish in the embryonal gut, it is likely that it will resemble a non-avian microbiome, such as a human (skin) microbiome, and not an avian gut/cloacal microbiome. Given the good hatchability and high similarity found between hatchling and female microbiomes (see Results Section), we rule out this source of contamination.

### 2.3. Sample Collection

On day 16 of incubation, we replaced the eggs with dummy eggs and transferred them to an incubator (37.5 °C, humidity >75%) in our indoor facility. We checked the incubator every 4 h between 9:00 and 21:00. When a chick had hatched, its first feces were collected using a sterile viscose swab (COPAN Diagnostics, Murrieta, CA, USA) but only if the feces were found inside the eggshell; this occurred in 87.6% of the 105 cases. The feces were placed in a sterilized vial and stored in a fridge (4–7 °C, maximal 1 h). We added a drop of sterilized sodium phosphate buffer (PBS) before storage at −20 °C until analysis. We analyzed feces of 16 randomly selected control and 17 randomly selected testosterone neonates.

We analyzed cloacal swabs of six randomly selected females, who due to the randomized design were not the biological mothers of the neonates but part of the group of females that incubated the eggs. The cloacal swabs were collected after the experiment had finished (23 August 2013), as adults are sensitive to handling stress during incubation and chick care. A sterile viscose swab was inserted into the cloaca without contacting feathers or skin, and gently rotated for 10 s in the intestinal lumen. We stored the tip of the swab in a sterilized 1.5 mL vial using a 76% ethanol sterilized clipper, and after adding a drop of sterile PBS, stored the swabs at −20 °C until analysis. We collected cloacal swabs from females because feces are not always produced during handling.

### 2.4. DNA Isolation, Amplification, and Sequencing

We isolated DNA from the samples using the Powersoil^®^ DNA kit (MoBio Laboratories, Carlsbad, CA, USA) according to the manufacturer’s instructions with the exception that we added extra sterile glass beads (~0.25 g) to the PowerBead tubes, and bead-beat the sample three times for one minute instead of three minutes continuously to prevent the sample from heating up too much (mini bead beater, BioSpec Products, Bartsville, OK, USA). We added the complete feces to the PowerBead tube, and from the swabs the fiber material plus cloacal material. Samples were randomized prior to DNA isolation. One negative control consisting of the fiber material of an unused, sterile swab was included in the DNA isolation procedure. Isolated DNA was stored at −20 °C until further use.

We quantified DNA concentrations in random order with the Quant-it PicoGreen dsDNA kit (Molecular Probes, Invitrogen, Eugene, OR, USA) to normalize the DNA concentrations to 1 ng template DNA per 25 μL reaction in the subsequent PCR. We randomized the samples again before amplifying the V4/V5 region of the 16S rRNA gene in a triplicate reaction using the primers 515F and 926R [25,26] with Illumina adaptors at the 5′-end. The thermal cycling protocol was: 5 min at 95 °C, 35 cycles with 40 s at 95 °C, 45 s at 56 °C, 40 s at 72 °C, followed by 10 min at 72 °C. Not all samples amplified during the PCR, leaving 13 control neonates, 15 testosterone neonates, six females, the negative control of the DNA isolation, and 4 negative PCR controls for downstream analysis. We sent the samples to GenoToul (INRA, Toulouse, France) for library preparations and Illumina sequencing using 2 × 250 bp v2 chemistry. For more details see [27].

### 2.5. Sequence Data Processing

We processed raw sequence data using QIIME v1.9.0 [28]. At GenoToul, sequence reads were demultiplexed and quality filtered using the default settings in QIIME. We joined paired-end reads and truncated reverse primers from joined reads. We used an open-reference OTU picking strategy with default QIIME settings using the Greengenes reference database (v13.8) [29] with de novo clustering of non-matching sequences (0.01%) using 97% identity in UCLUST [30]. We selected representative sequences for all OTUs prior to merging both OTU tables. Singletons were removed, taxonomy assigned with UCLUST (Greengenes v13.8, 97%), and representative sequences were aligned using PyNast (default settings [31]). After removing chimeric sequences with UCHIME [32], we constructed the phylogenetic tree (FastTree [33]). Next, we filtered archaea, chloroplasts, and mitochondria from the OTU table. To avoid bias of contamination of the DNA extraction and subsequent lab-procedures [34], we conservatively removed all 311 OTUs present in the negative controls from the data set, including the singletons (a taxa table is provided in the supplementary information, Table S1, as well as rarefaction curves and relative abundances of classes, Figure S1). As not all 311 OTUs found in the negative controls were present in the samples, this excluded 258 OTUs from the sample data. Hereafter, the total number of sequence reads was 128,060.

### 2.6. Statistical Analysis

We analyzed bacterial diversity in R (v3.4.3 [35]) using the R packages *Phyloseq* (v1.22.3 [36]), *vegan* (v2.4-4 [37]), and *DESeq2* (v1.18.0 [38]). To check for outliers, we plotted the mean abundance per OTU in neonates versus the mean abundance per OTU in females (supplementary information Figure S2a). This figure showed two outlier OTUs in neonates, namely two *Staphylococcus* (g). These OTUs were the two most abundant OTUs in neonates; their abundances were 7–14.5 times higher than the third most abundant OTU. These high abundances were due to only three of the 28 neonates (one control and two testosterone neonates), who together accounted for 98.4% of the total abundance of these OTUs. Because our samples may be sensitive to possible sample processing problems due to the relatively low DNA concentrations [34], we conservatively removed these three neonate samples from the data. Note that these two OTUs were not removed from the data set.

Rarefaction curves of the remaining samples showed that OTU richness had not reached saturation, while the Shannon diversity levelled off around 1000 reads (supplementary information Figure S2c). However, due to low sequencing depth of the neonate samples and to prevent severe sample loss, we did not rarefy the data. Instead we analyzed the samples within the highest 90% sequencing depth to increase power and information density. This left a data set consisting of 5 females, 9 control, and 12 testosterone neonates. Lastly, we removed singletons, leaving 66,666 sequence reads, resulting in a total of 2881 OTUs, divided over 254 genera, 137 families, 64 orders, and 15 phyla. For analyses at phylum-level, we divided the Proteobacteria phylum into its five classes present, resulting thus in 19 taxonomic groups, hereafter referred to as “phylum” or “phyla”.

Comparison between control and testosterone neonates revealed marginal differences: relative abundance differed in only one “phylum” (Firmicutes), one order (Rhodobacterales, Alphaprotebacteria), one genus (*Paracoccus*, Alphaproteobacteria), and only one OTU (unassigned *Atopobium* genus, Actinobacteria) (*ANCOM* false discovery rate corrected (FDR) *q* < 0.05); while DESeq2-corrected read counts did not differ at any taxonomic level (*DESeq2* FDR *q* > 0.1); and finally unweighted and weighted UniFrac distances and Bray−Curtis dissimilarities did not differ (PERMANOVA *p* = 0.18, *r*^2^ = 0.06, *p* = 0.39, *r*^2^ = 0.05, and *p* = 0.05, *r*^2^ = 0.06, respectively). We therefore combined neonatal groups in the analyses and compared them as one group with the females.

Because the data were not rarefied, we could not analyze alpha-diversity, as these measures are strongly correlated with the number of reads. Rank abundance plots (supplementary information Figure S2b) showed comparable patterns in neonates and females, with a few very abundant OTUs and many rare OTUs. We assessed differences in absolute abundance at “phylum”-, order-, genus-, and OTU-level in *DESeq2* using negative binomial models and Wald-test (critical false discovery rate corrected (FDR) *q*-value 0.1). Differences in relative abundance between groups at “phylum”-, order-, genus-, and OTU-level were assessed using an analysis of the composition of microbiomes (*ANCOM*) [39], with a critical FDR *q*-value of 0.05. We characterized the core microbiome in neonates and females separately using the microbiome package (v1.0.2 [40]). OTUs were included in the core microbiome when present in 60% of the samples. We visualized and compared co-occurrence of OTUs between females and neonates, and within females, with Venn diagrams (venn v1.2 [41]).

Read counts were transformed in *DESeq2* (variance stabilizing transformation) before assessing phylogenetic similarities between groups in bacterial community composition (beta-diversity) using unweighted (community membership: presence/absence table) and weighted UniFrac distances (community structure: presence/absence/abundance matrix [42]) and taxonomic similarities between groups using Bray−Curtis dissimilarities. Prior to this analysis, we corrected the data for the negative values remaining after the variance stabilizing transformation by adding first the negative value of the originally zero abundance to the data (up to the fourth decimal, so that a very small negative value remained) and then converting all negative values to zero. We performed a principal coordinate ordination analysis (PCoA) of the distances/dissimilarities using vegan. We tested if community clustering and group dispersion (i.e., mean distance to the cluster centroid to represent the variation among individuals within a group) differed between neonates and females, by modelling unweighted and weighted UniFrac distances, and Bray−Curtis dissimilarities from an OTU-level table using PERMANOVA with 999 permutations (ADONIS function in vegan) [43,44]. We used the ‘betadisper’ function [45] in vegan to evaluate the degree of within-group dispersions (permutest; a non-significant *p*-value indicates that the difference found is not due to differences in groups dispersions). Permutests were not significant for unweighted and weighted UniFrac comparisons (*p* = 0.13 and *p* = 0.17, respectively), but it was significant for the Bray−Curtis comparison (*p* = 0.004). However, plots of group dispersions were very similar between beta-measures (supplementary information Figure S5c−e).

Finally, we calculated pair-wise unweighted and weighted UniFrac distances, and Bray−Curtis dissimilarities for each neonate−female combination. Pair-wise distances/dissimilarities were not normally distributed (Shapiro−Wilk normality test, *W* = 0.93, *p* <0.001), so we tested for differences using a Kruskal−Wallis test with a post-hoc two-sided Dunn test (Bonferoni, *FSA* v.0.8.19 [46]).

### 2.7. Comparison with Published Data

Jinmei Ding and co-authors [20], kindly provided us with the abundance of and taxonomic data on adult and neonate chickens. We treated the data similar to our data and removed singletons before variance stabilizing transforming read counts in *DESeq2*. We corrected the data for negative values as described above prior to calculating unweighted and weighted UniFrac distances and Bray−Curtis dissimilarities. We calculated pair-wise unweighted and weighted UniFrac distances, and Bray−Curtis dissimilarities for each female (*n* = 12) and 4-day-old chicken embryo (*n* = 27) combination and for each female and 19-day-old chicken embryo (*n* = 24) combination. Note that the pair-wise distances did not differ between embryo ages (Kruskal−Wallis χ^2^ = 2.30, *df* = 1, *p* = 0.13).

Chu and co-authors [47] and Collado and co-authors [48] kindly provided us with matrixes of pair-wise unweighted and weighted UniFrac distances [47,48] and Bray−Curtis dissimilarities [47] between female stool and neonate meconium samples. To enable comparison on the population level, we did not select for mother−child pair-wise distances but instead included all pair-wise distances of each females−neonate combination before calculating mean distances (overall *n* = 7488 and *n* = 195 for [47,48], respectively).

## 3. Results

**Figure 1:**
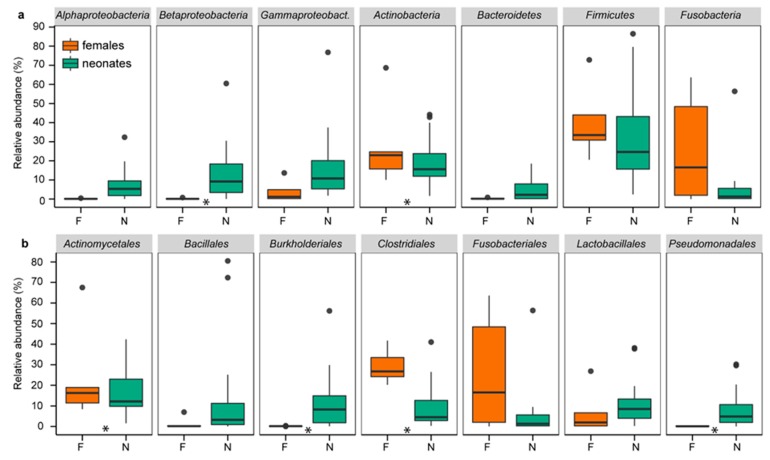
Relative abundances of the most common “phyla” (**a**) and orders (**b**) in neonates and females. Relative abundances of the presented “phyla” and orders were >1% and >5%, respectively. Boxplots present the median, 25^th^ and 75^th^ percentiles and, if applicable, outliers. Green boxes are neonates (N). Orange boxes are females (F). Stars indicate significant differences between neonates and females.

Similar to chickens, rats, Japanese macaques, and humans [2,8,16,17,20], the first feces of neonatal rock pigeons contained a well-established microbiome resembling the female cloacal microbiome, as indicated by the multiple shared “phyla”, orders, families, and genera (Figure 1, supplementary information Figure S3). The four phyla commonly found in birds, Actinobacteria, Bacteroidetes, Firmicutes, and Proteobacteria [49,50], were among the five most abundant phyla in neonates (16.1%, 4.1%, 36.9%, and 36.4%, respectively) and females (19.2%, 0.2%, 33.6%, and 5.5%, respectively).

Next, we compared DESeq2 transformed abundances and relative abundances at various taxonomic levels to determine the degree of similarity of neonatal and female bacterial communities. Abundances differed only in 3 of the 15 “phyla” (Alphaproteobacteria, Betaproteobacteria, and Bacteroidetes), 15 of the 64 orders, and 20 of the 254 genera (*DESeq2*, FDR *q* < 0.1; supplementary information Figure S4, Table S2), and not at OTU level. Relative abundances similarly differed in only 2 “phyla” (Actinobacteria and Betaproteobacteria), 6 orders, 3 genera, and 15 of the 2881 OTUs (*ANCOM*, FDR *q* < 0.05; supplementary information Table S2). Neonates and females shared thus multiple “phyla”, orders, families, and genera, with few differences in DESeq2 transformed abundances and relative abundances.

**Figure 2:**
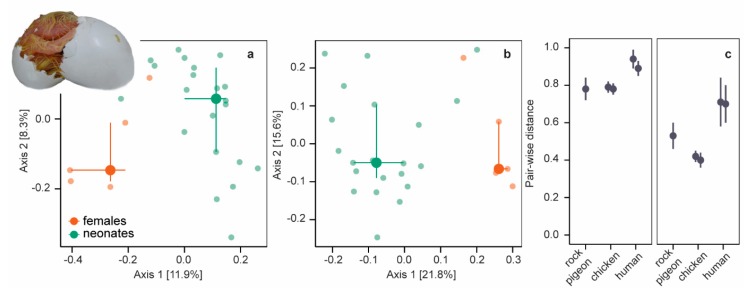
Unweighted and weighted Unifrac distances. (**a**) PCoA plot of unweighted UniFrac distances of neonates (green symbols) and females (orange symbols). Large symbols present medians, the error bars the 25% and 75% quantiles. Transparent symbols present the underlying data. (**b**) PCoA plot of weighted UniFrac distances. (**c**) Mean pair-wise unweighted (left panel) and weighted UniFrac (right panel) distances (± SD) between embryos/neonates and females in five datasets: rock pigeon (*n* = 105 [this study]), chicken after incubation periods of 4 days (*n* = 324) and 19 days (*n* = 288) [20], and humans (*n* = 7488 [47] and *n* = 195 [48]).

We further explored (dis)similarities between neonatal and female bacterial communities by comparing phylogenetic (unweighted and weighted UniFrac distances) and taxonomic (Bray−Curtis dissimilarities) composition using principal coordinate ordination analysis (PCoA) of variance stabilizing transformed abundances. Unweighted and weighted UniFrac distances (Figure 2a,b) and Bray−Curtis dissimilarities (supplementary information Figure S5a) differed between neonates and females (all PERMANOVA *p* = 0.001, *r^2^* < 0.15). Phylogenetic and taxonomic community composition differed also between neonatal and female microbiomes in chickens [20] and mammals [17,47,48,51] (but see [52]) and seems a common phenomenon.

For a more detailed comparison, we calculated pair-wise beta-diversity dissimilarities/distances for each neonate−female combination and included chicken (embryos of two ages) and human data (two studies) in our analyses. Pair-wise dissimilarities/distances differed between beta-diversity measures in all species (Kruskal−Wallis *χ*^2^ = 17017, *df* = 2, *p* < 0.001, post-hoc two-sided Dunn-test all *P_adj_* < 0.001; species combined): pairwise Bray−Curtis dissimilarities and unweighted UniFrac distances were large, whereas pair-wise weighted UniFrac distances were smaller (Figure 2c, supplementary information Figure S5b). Small pair-wise weighted UniFrac distances indicate that neonatal and female microbiomes were more similar among the more abundant OTUs. Pair-wise beta-diversity dissimilarities/distances were smallest in rock pigeon and chickens (Kruskal−Wallis all χ^2^ > 1668, *df* = 1, *p* < 0.001), indicating that neonatal microbiomes were more equivalent to female microbiomes in birds than in humans.

Neonatal rock pigeons shared 45.5% of their core microbiome (11 OTUs, present in 60% of the samples) with that of females (28 OTUs; supplementary information Table S3), namely an unassigned Leptotrichiaceae family OTU, an unassigned *Anaerococcus* genus, *Veillonella dispar*, and two unassigned *Dialister* genus OTUs. Two core microbiome OTUs belonged to the top 10 most abundant OTUs in neonates: the unassigned Leptotrichiaceae family OTU (2.2%) and *Staphylococcus* genus OTU (1.1%). In females, eight core microbiome OTUs belonged to their top 10 most abundant OTUs, among which the unassigned Leptotrichiaceae family OTU, which was the most abundant OTU in females (35.6%). Leptotrichiaceae (Fusobacteria) are obligatory anaerobic bacteria that colonize mucous membranes in oral cavities, intestines, and urogenital tracts [53].

**Figure 3:**
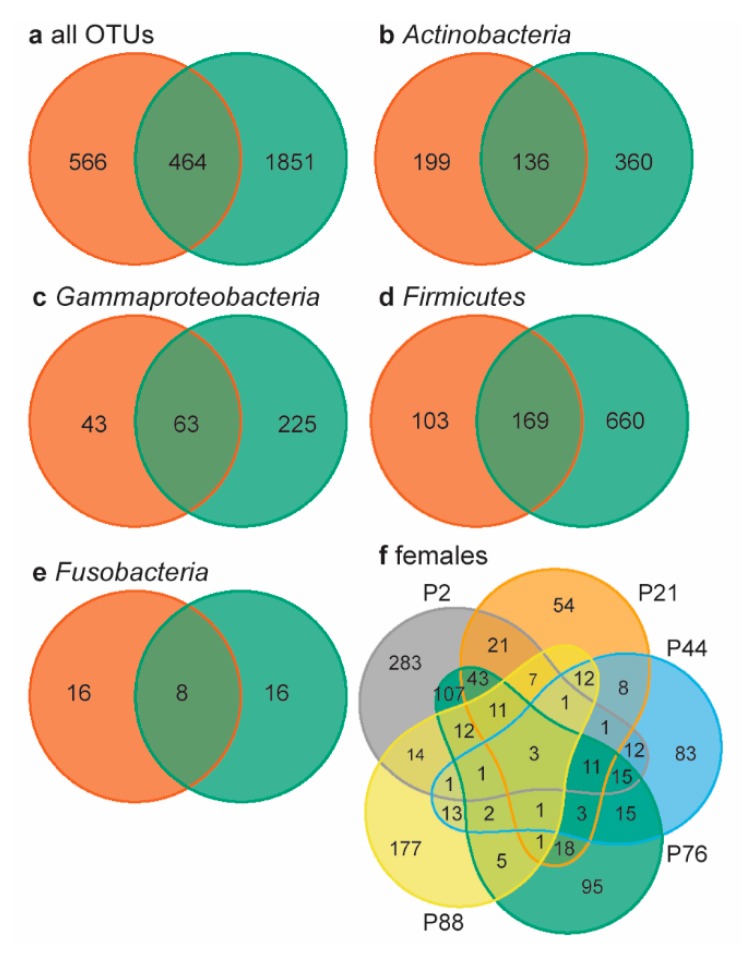
Venn diagrams of neonate and female microbiomes. (**a**) Females (orange) and neonates (green) shared 16.1% of the 2881 OTUs present in all samples. In the four most abundant phyla, neonates and females shared 19.6% of the OTUS within *Actinbacteria* ((**b**), 17.3% total relative abundance), 19.0% within *Gammaproteobacteria* ((**c**), 10.7% total relative abundance), 18.1% within *Firmicutes* ((**d**), 35.6% total relative abundance), and 20.0% within *Fusobacteria* ((**e**), 18.4% total relative abundanc). (**f**) The five females shared 3 OTUs, i.e., 0.3% of all female OTUs. Individual females are indicated by color and *p*-number.

Lastly, we determined the overall number of shared OTUs using Venn diagrams, by looking beyond the core microbiome. Neonates and females shared 16.1% of the 2881 OTUs present in all samples (Figure 3a). Within the four most abundant bacterial “phyla” (overall relative abundance >10%), neonates and females shared 18−20% of the OTUs present (Figure 3b−e). In chickens, embryos and females shared 35.8% of the genera present [20]; rock pigeons neonates similarly shared 32.7% of the genera with females. The percentage of shared OTUs between neonates and females was remarkably high, considering the large inter-individual variation in female microbiomes: females shared only three of the 1030 OTUs present (0.3%, Figure 3f). This very low percentage of shared OTUs may be related to the large number of rare OTUs in our data. However, large individual variation in gut microbiomes is also common [54]. Consequently, the relatively high percentage of shared OTUs between neonates and females is a strong indication that neonates obtained gut bacteria through prenatal transfer.

## 4. Discussion

The first feces of rock pigeons hatchlings contained a well-established avian microbiome. Hatchlings produced their first feces immediately upon hatching before leaving the eggshell in an adult-free environment, thus prior to the onset of post-hatch bacterial colonization due to horizontal transfer from the environment or eggshell or postnatal transfer from food or parents. Colonization from the environment, food or parents can thus not explain the high similarity between hatchling and female microbiomes. The multiple shared taxa, few differences in (relative) abundances, and the high similarities between the core microbiomes of hatchlings and females therefore suggest that prenatal maternal gut bacteria transfer does occur in rock pigeons, which is consistent with the findings in chickens [20] and mammals [2,8,15,16,17,20]. In addition, recent indications for prenatal transfer of gut bacteria to eggs were found in four wild passerine bird species and the wild eastern fence lizard (*Sceloporus undulates*) [55].

Avian gut bacteria have several options to migrate to eggs. Firstly, bacteria attached to the egg could penetrate it through the egg pores, despite the egg’s protective physical and chemical barriers [56,57,58,59]. However, egg viability studies on wild birds have shown that the rate of microbial penetration is low in a temperate environment [60], while incubation withstands microbial penetration [57,61,62,63]. Moreover, eggshell microbiomes of freshly laid eggs of woodlarks (*Lullula arborea*) and skylarks (*Alauda arvensis*) did not resemble maternal cloacal microbiomes [64]. It is possible though, that bacteria penetrate the eggshell pre-laying in the vagina [56].

Secondly, bacteria may invade the oviduct—and from there all egg compartments—directly from the cloaca, where the oviduct flows out [56]. In humans, vaginal bacteria have been shown to invade the placenta and amniotic fluids [2,8] indicating the movement potential of bacteria. Thus, bacteria from higher up in the intestinal tract may potentially also use the cloacal route to invade the oviduct, as gut bacteria can be mobile within a gut [65,66] and may benefit from downstream transfer with the gut contents to reach the cloaca.

Thirdly, gut bacteria may migrate to the oviduct by invading the bloodstream through uptake by macrophages that invade the intestines, as shown in *Salmonella* [56]. A similar mechanism is found in mammals where dendritic cells actively penetrate the gut epithelium to take up bacteria [8,10]. And lastly, penetration of the ovary may be possible, just as *Salmonella* can attach to or invade developing and mature follicular granulosa cells [56].

In our study, we compared fecal microbiomes of hatchlings with cloacal microbiomes of adult females, which represent different portions of the gut microbiome [67]. This may result in differences between fecal and cloacal microbiomes, making our comparison conservative. Nevertheless, neonatal and female microbiomes were more similar in rock pigeons and chickens than in humans. This may occur if in birds penetration via the cloaca is the major route for gut bacteria to invade the oviduct. Then mainly bacteria inhabiting the cloaca and nearby gut compartments are expected to invade the egg, resulting in a closer resemblance between avian embryonic/neonatal fecal and female cloacal microbiomes. Alternatively, the lower resemblance in humans may be explained by the fact that in humans neonatal gut bacteria originate from various female gut compartments, including the oral cavity, as well as the vagina [2,8]. Since pair-wise dissimilarities/distances were calculated between meconium and female stool samples, neonatal bacteria originating from the vagina or distant gut compartments such as the oral cavity may increase the pair-wise distances/dissimilarities found.

## 5. Conclusions

Overall, our study shows that prenatal transfer of gut bacteria does occur in birds, supporting our hypothesis that prenatal transfer occurs in oviparous vertebrates and confirming findings in chicken [20], passerines, and fence lizards [55]. Hence, a continuous maternal transfer during embryonic development, as may occur during pregnancy in mammals [2,8,10,15,16,17], is not required for the prenatal transfer of gut bacteria in vertebrates. Our results fuel the expectation that prenatal maternal transfer of gut bacteria is universal in the animal kingdom, which is beneficial for both fetal and postnatal development [2,8,10]. Prenatal transfer of gut bacteria indicates that the bacterial component of the gut microbiome can be considered as an inheritable trait, passed on from one generation to another. Our study shows the potential for non-invasive studies on prenatal transfer in wild, free-living oviparous vertebrates on a larger scale, as the neonatal first feces can be compared with female samples randomly collected from the population. Further studies are needed to verify whether these findings extend to other components of the maternal microbiome (fungi, viruses, protozoans) and the functions these organisms provide to the host.

## Supplementary Materials

The following are available online at https://www.mdpi.com/2076-2607/8/1/61/s1, Figure S1: Rarefaction curves and relative abundances of classes of the five negative control samples, Figure S2: Mean abundance per OTU, rank abundance plots, and rarefaction curves, Figure S3: The relative abundances of the most common families and genera (i.e., relative abundance >1%), Figure S4: Significant differences in *DESeq2* transformed abundances (FDR *q* < 0.1) between neonates and females at “phylum”, order, and genus level, Figure S5: Bray–Curtis distances of rock pigeon and literature data and group dispersion plots of all beta-diversities, Table S1: Overview of the 311 OTUs present in the five negative control samples, Table S2: Overview of phyla, orders, genera, and OTUs differing in normalized (*DESeq2*) or relative abundance, Table S3: Overview of the OTUs present in core microbiome of neonates and females. The processed dataset supporting the conclusions of this article is included in the supplementary information. The QIIME script is available as supplementary information Script S1 (TXT file). The R-script is available as supplementary information Script S2 (R file). The OTU community table file of the female and neonate samples for the creation of a *phyloseq* object, with the OTUs of the negative controls removed from the data set, is available as supplementary information Biom S1 (json format BIOM file). The phylogenetic tree file for analysis in R is available as supplementary information Tree S1 (TRE file). The metadata for the creation of a *phyloseq* object are given in supplementary information Data S1 (XLSX file). The key to the column names of the metadata file is provided in supplementary information Data S2 (DOCX file). Finally, the data used for the analysis of the pairwise beta-diversity distances and dissimilarities is provided in supplementary information Data S3 (XLXS file). The raw sequence datasets (Fasta files) generated and/or analyzed in the current study are available in the DataverseNL database via the public accessible persistent link https://dataverse.nl/dataset.xhtml?persistentId=hdl:10411/IMAK0Q.

## References

[1] McFall-Ngai M., Hadfield M.G., Bosch T.C.G., Carey H.V., Domazet-Lošo T., Douglas A.E., Dubilier N., Eberl G., Fukami T., Gilbert S.F. (2013). Animals in a bacterial world, a new imperative for the life sciences. Proc. Natl. Acad. Sci. USA.

[2] Walker R.W., Clemente J.C., Peter I., Loos R.J.F. (2017). The prenatal gut microbiome: Are we colonized with bacteria in utero?. Pediatr. Obes..

[3] Kohl K.D. (2012). Diversity and function of the avian gut microbiota. J. Comp. Physiol. B.

[4] Rees T., Bosch T., Douglas A.E. (2018). How the microbiome challenges our concept of self. PLoS Biol..

[5] Macke E., Tasiemski A., Massol F., Callens M., Decaestecker E. (2017). Life history and eco-evolutionary dynamics in light of the gut microbiota. Oikos.

[6] Lee Y.K., Mazmanian S.K. (2010). Has the microbiota played a critical role in the evolution of the adaptive immune system?. Science.

[7] Davenport E.R., Sanders J.G., Song S.J., Amato K.R., Clark A.G., Knight R. (2017). The human microbiome in evolution. BMC Biol..

[8] Stinson L.F., Payne M.S., Keelan J.A. (2017). Planting the seed: Origins, composition, and postnatal health significance of the fetal gastrointestinal microbiota. Crit. Rev. Microbiol..

[9] Cox L.M., Yamanishi S., Sohn J., Alekseyenko A.V., Leung J.M., Cho I., Kim S.G., Li H., Gao Z., Mahana D. (2014). Altering the intestinal microbiota during a critical developmental window has lasting metabolic consequences. Cell.

[10] Funkhouser L.J., Bordenstein S.R. (2013). Mom knows best: The universality of maternal microbial transmission. PLoS Biol..

[11] Gensollen T., Iyer S.S., Kasper D.L., Blumberg R.S. (2016). How colonization by microbiota in early life shapes the immune system. Science.

[12] Nylund L., Satokari R., Salminen S., De Vos W.M. (2014). Intestinal microbiota during early life-Impact on health and disease. Proc. Nutr. Soc..

[13] Romano-Keeler J., Weitkamp J.H. (2015). Maternal influences on fetal microbial colonization and immune development. Pediatr. Res..

[14] Bright M., Bulgheresi S. (2010). A complex journey: Transmission of microbial symbionts. Nat. Rev. Microbiol..

[15] Jiménez E., Marín M.L., Martín R., Odriozola J.M., Olivares M., Xaus J., Fernández L., Rodríguez J.M. (2008). Is meconium from healthy newborns actually sterile?. Res. Microbiol..

[16] Borghi E., Massa V., Severgnini M., Fazio G., Avagliano L., Menegola E., Bulfamante G., Morace G., Borgo F. (2017). Antenatal microbial colonisation of mammalian gut. bioRxiv.

[17] Chu D., Prince A., Ma J., Pace R., Takahashi D., Friedman J., Kievit P., Sullivan E., Grove K., Aagaard K. (2018). Contribution of the fetal microbiome to the taxonomic diversity and functionality of the postnatal gut microbiome in a non-human primate (NHP) model. Am. J. Obs. Gynecol..

[18] Leblois J., Massart S., Li B., Wavreille J., Bindelle J., Everaert N. (2017). Modulation of piglets’ microbiota: Differential effects by a high wheat bran maternal diet during gestation and lactation. Sci. Rep..

[19] Grond K., Lanctot R.B., Jumpponen A., Sandercock B.K. (2017). Recruitment and establishment of the gut microbiome in arctic shorebirds. FEMS Microbiol. Ecol..

[20] Ding J., Dai R., Yang L., He C., Xu K., Liu S., Zhao W., Xiao L., Luo L., Zhang Y. (2017). Inheritance and establishment of gut microbiota in chickens. Front. Microbiol..

[21] Pedroso A.A., Batal A.B., Lee M.D. (2016). Effect of in ovo administration of an adult-derived microbiota on establishment of the intestinal microbiome in chickens. Am. J. Vet. Res..

[22] Hsu B.-Y.Y., Dijkstra C., Groothuis T.G.G.G. (2016). No escape from mother’s will: Effects of maternal testosterone on offspring reproductive behaviour far into adulthood. Anim. Behav..

[23] Hsu B. (2016). Maternal Hormones Meet Environmental Variability. Context-Dependent Effects of Maternal Hormones in Avian Egg Yolks. Ph.D. Thesis.

[24] Hetmański T., Barkowska M. (2007). Density and age of breeding pairs influence feral pigeon, *Columba livia* reproduction. Folia Zool..

[25] Parada A.E., Needham D.M., Fuhrman J.A. (2016). Every base matters: Assessing small subunit rRNA primers for marine microbiomes with mock communities, time series and global field samples. Environ. Microbiol..

[26] Quince C., Lanzen A., Davenport R.J., Turnbaugh P.J. (2011). Removing Noise From Pyrosequenced Amplicons. BMC Bioinform..

[27] van Veelen H.P.J., Falcao Salles J., Tieleman B.I. (2017). Multi-level comparisons of cloacal, skin, feather and nest-associated microbiota suggest considerable influence of horizontal acquisition on the microbiota assembly of sympatric woodlarks and skylarks. Microbiome.

[28] Caporaso J.G., Kuczynski J., Stombaugh J., Bittinger K., Bushman F.D., Costello E.K., Fierer N., Peña A.G., Goodrich J.K., Gordon J.I. (2010). QIIME allows analysis of high-throughput community sequencing data. Nat. Methods.

[29] DeSantis T.Z., Hugenholtz P., Larsen N., Rojas M., Brodie E.L., Keller K., Huber T., Dalevi D., Hu P., Andersen G.L. (2006). Greengenes, a chimera-checked 16S rRNA gene database and workbench compatible with ARB. Appl. Environ. Microbiol..

[30] Edgar R. (2010). Search and clustering orders of magnitude faster than BLAST. Bioinformatics.

[31] Caporaso J.G., Bittinger K., Bushman F.D., DeSantis T.Z., Anderson G.L., Knight R. (2010). PyNast: A flexible tool of chimera detection. Bioinformatics.

[32] Edgar R.C., Haas B.J., Clemente J.C., Quince C., Knight R. (2011). UCHIME improves sensitivity and speed of chimera detection. Bioinformatics.

[33] Price M.N., Dehal P.S., Arkin A.P. (2009). FastTree: Computing large minimum evolution trees with profiles instead of a distance matrix. Mol. Biol. Evol..

[34] Salter S.J., Cox M.J., Turek E.M., Calus S.T., Cookson W.O., Moffatt M.F., Turner P., Parkhill J., Loman N.J., Walker A.W. (2014). Reeagent and labaratory contamination can critically impact sequence-based microbiome analyses. BMC Biol..

[35] R Core Team R: A Language and Environment for Statistical Computing. http://www.r-project.org.

[36] McMurdie P.J., Holmes S. (2013). phyloseq: An R package for reproducible interactive analysis and graphics of microbiome census data. PLoS ONE.

[37] Oksanen J., Blanchet F.G., Kindt R., Legendre P., Minchin P.R., Hara R.B.O., Simpson G.L., Solymos P., Stevens M.H.H., Wagner H. Vegan: Community Ecology Package. https://cran.r-project.org/package=vegan.

[38] Love M.I., Huber W., Anders S. (2014). Moderated estimation of fold change and dispersion for RNA-seq data with DESeq2. Genome Biol..

[39] Mandal S., Van Treuren W., White R.A., Eggesbø M., Knight R., Peddada S.D. (2015). Analysis of composition of microbiomes: A novel method for studying microbial composition. Microb. Ecol. Heal. Dis..

[40] Lahti L., Shetty S., Blake T., Salojarvi J. Microbiome R Package. http://bioconductor.org/packages/microbiome/.

[41] Dusa A. Draw Venn Diagrams. https://cran.r-project.org/web/packages/venn/index.html.

[42] Lozupone C., Knight R. (2005). UniFrac: A new phylogenetic method for comparing microbial communities. Appl. Environ. Microbiol..

[43] Anderson M.J. (2001). A new method for non-parametric multivariate analysis of variance. Austral Ecol..

[44] McArdle B.H., Anderson M.J. (2001). Fitting multivariate models to community data: A comment on distance-based redundancy analysis. Ecology.

[45] Anderson M.J. (2006). Distance-based tests for homogeneity of multivariate dispersions. Biometrics.

[46] Ogle D.H. FSA: Fisheries Stock Analysis. https://cran.r-project.org/web/packages/FSA/index.html.

[47] Chu D.M., Ma J., Prince A.L., Antony K.M., Seferovic M.D., Aagaard K.M. (2017). Maturation of the infant microbiome community structure and function across multiple body sites and in relation to mode of delivery. Nat. Med..

[48] Collado M.C., Rautava S., Aakko J., Isolauri E., Salminen S., Xiii I.S., Xi I.S. (2016). Human gut colonisation may be initiated in utero by distinct microbial communities in the placenta and amniotic fluid. Sci. Rep..

[49] Waite D.W., Taylor M.W. (2014). Characterizing the avian gut microbiota: Membership, driving influences, and potential function. Front. Microbiol..

[50] Hird S.M., Sánchez C., Carstens B.C., Brumfield R.T. (2015). Comparative gut microbiota of 59 neotropical bird species. Front. Microbiol..

[51] Gosalbes M.J., Llop S., Vallès Y., Moya A., Ballester F., Francino M.P. (2013). Meconium microbiota types dominated by lactic acid or enteric bacteria are differentially associated with maternal eczema and respiratory problems in infants. Clin. Exp. Allergy.

[52] Hu J., Nomura Y., Bashir A., Fernandez-Hernandez H., Itzkowitz S., Pei Z., Stone J., Loudon H., Peter I. (2013). Diversified microbiota of meconium is affected by maternal diabetes status. PLoS ONE.

[53] Eisenberg T., Fawzy A., Nicklas W., Semmler T., Ewers C. (2016). Phylogenetic and comparative genomics of the family Leptotrichiaceae and introduction of a novel fingerprinting MLVA for *Streptobacillus moniliformis*. BMC Genom..

[54] Shapira M. (2016). Gut microbiotas and host evolution: Scaling up symbiosis. Trends Ecol. Evol..

[55] Trevelline B.K., MacLeod K.J., Knutie S.A., Langkilde T., Kohl K.D. (2018). In ovo microbial communities: A potential mechanism for the initial acquisition of gut microbiota among oviparous birds and lizards. Biol. Lett..

[56] Gantois I., Ducatelle R., Pasmans F., Haesebrouck F., Gast R., Humphrey T.J., Van Immerseel F. (2009). Mechanisms of egg contamination by *Salmonella enteritidis*. FEMS Microbiol. Rev..

[57] Cook M.I., Beissinger S.R., Toranzos G.A., Arendt W.J. (2005). Incubation reduces microbial growth on eggshells and the opportunity for trans-shell infection. Ecol. Lett..

[58] Cook M.I., Beissinger S.R., Toranzos G.A., Rodriguez R.A., Arendt W.J. (2005). Microbial infection affects egg viability and incubation behavior in a tropical passerine. Behav. Ecol..

[59] Cook M.I., Beissinger S.R., Toranzos G.A., Rodriguez R.A., Arendt W.J. (2003). Trans-shell infection by pathogenic micro-organisms reduces the shelf life of non-incubated bird’s eggs: A constraint on the onset of incubation?. Proc. R. Soc. B Biol. Sci..

[60] Wang J.M., Firestone M.K., Beissinger S.R. (2011). Microbial and environmental effects on avian egg viability: Do tropical mechanisms act in a temperate environment?. Ecology.

[61] Shawkey M.D., Firestone M.K., Brodie E.L., Beissinger S.R. (2009). Avian incubation inhibits growth and diversification of bacterial assemblages on eggs. PLoS ONE.

[62] D’Alba L., Oborn A., Shawkey M.D. (2010). Experimental evidence that keeping eggs dry is a mechanism for the antimicrobial effects of avian incubation. Naturwissenschaften.

[63] Ruiz-de-castañeda R., Vela A.I., Lobato E., Briones V., Moreno J. (2012). Early onset of oncubation and eggshell bacterial loads in a temperate-zone cavity-nesting passerine. Condor.

[64] van Veelen H.P.J., Salles J.F., Tieleman B.I.I. (2018). Microbiome assembly of avian eggshells and their potential as transgenerational carriers of maternal microbiota. ISME J..

[65] Obadia B., Güvener Z.T., Zhang V., Ceja-Navarro J.A., Brodie E.L., Ja W.W., Ludington W.B. (2017). Probabilistic invasion underlies natural gut microbiome stability. Curr. Biol..

[66] Randal Bollinger R., Barbas A.S., Bush E.L., Lin S.S., Parker W. (2007). Biofilms in the large bowel suggest an apparent function of the human vermiform appendix. J. Theor. Biol..

[67] Videvall E., Strandh M., Engelbrecht A., Cloete S., Cornwallis C.K. (2018). Measuring the gut microbiome in birds: Comparison of faecal and cloacal sampling. Mol. Ecol. Resour..

