# Supplementary material for: Prenatal Transfer of Gut Bacteria in Rock Pigeon
Source: Microorganisms. 2019 Dec 30;8(1):61. doi: 10.3390/microorganisms8010061 (PMC7022786; doi:10.3390/microorganisms8010061)
Supplement: Supplementary file 1 [file microorganisms-08-00061-s001.zip › Dietz et al Data S2.docx]

**Data for:**

MW Dietz, JF Salles, B-Y Hsu, C Dijkstra, TGG Groothuis, M van der Velde, YI Verkuil, BI Tieleman: Prenatal transfer of gut bacteria in birds: evidence from rock pigeons

**Key to column names of** Dietz_et_al_2018_Prenatal_gut_bacteria_transfer_in_birds_evidence_from_rock_pigeons_Microbiome_Additional_file_6.txt; Text-file containing the metadata for use in R to create a *Phyloseq* object.

SampleID – Sample identification number given upon DNA extraction.

BarcodeSquence – Sequence of the illumina adapters used to identify the samples

LinkerPrimerSequence – Forward primer sequence

ReversePrimer – Reverse primer sequence

InputFileName – Sample file name after merging of the paired-end sequences.

Sampletype – Type of sample: FemaleCloaca – cloacal swab of adult female, HatchlingFirstFeces – neonatal first feces.

RPd0FClean – Grouping factor to exclude negative controls and outliers.

RPd0NC – Grouping factor to exclude negative controls.

BirdID – unique identifier of the bird.

ChickAdult – Whether the sample was from an adult female (AD) or neonate (CH).

Testosterone – Whether the sample was from a neonate hatched from a control egg (Control), testosterone injected egg (Testosterone) or adult female or negative control (NA).

DNAconc_post_PCR – The DNA concentration from the combined triplicate PCR products determined via nanodrop.
