# Supplementary material for: Prenatal Transfer of Gut Bacteria in Rock Pigeon
Source: Microorganisms. 2019 Dec 30;8(1):61. doi: 10.3390/microorganisms8010061 (PMC7022786; doi:10.3390/microorganisms8010061)
Supplement: Supplementary file 1 [file microorganisms-08-00061-s001.zip › Dietz et al Supplementary Information - Microorganisms.docx]

**Supplementary Information belonging to Dietz et al 2019 Prenatal transfer of gut bacteria in rock pigeon**

This file contains:

Figures S1 to S5

Tables S1 to S3


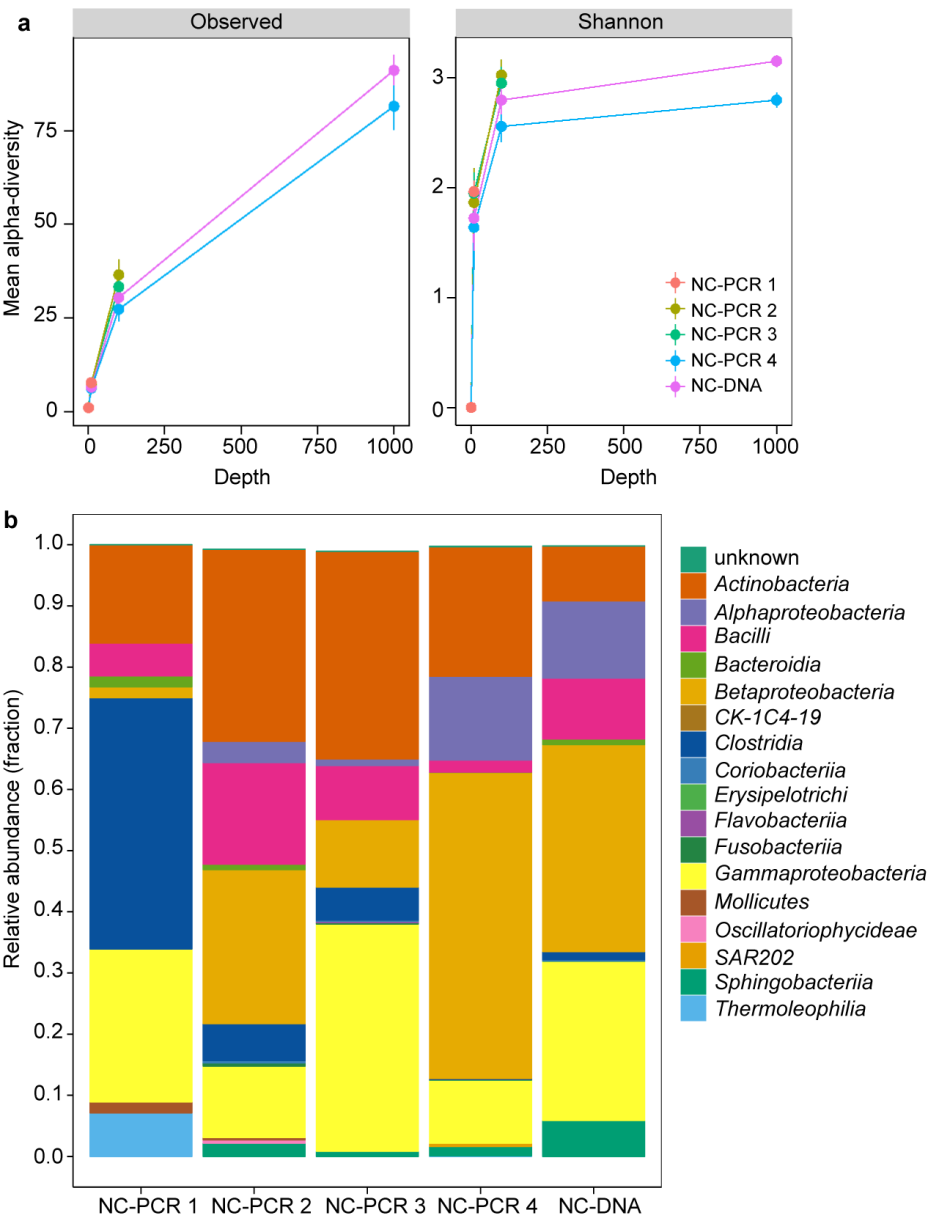


**Fig. S1.** Rarefaction curves (**a)** and relative abundances of classes (**b)** of the five negative control samples. NC-DNA is the negative control of the DNA extraction. The NC-PCRs are the negative controls of the first PCR. Note that NC-PCR 1 shows a different distribution of relative class abundances. In total 311 OTUs were present in the five negative control samples.


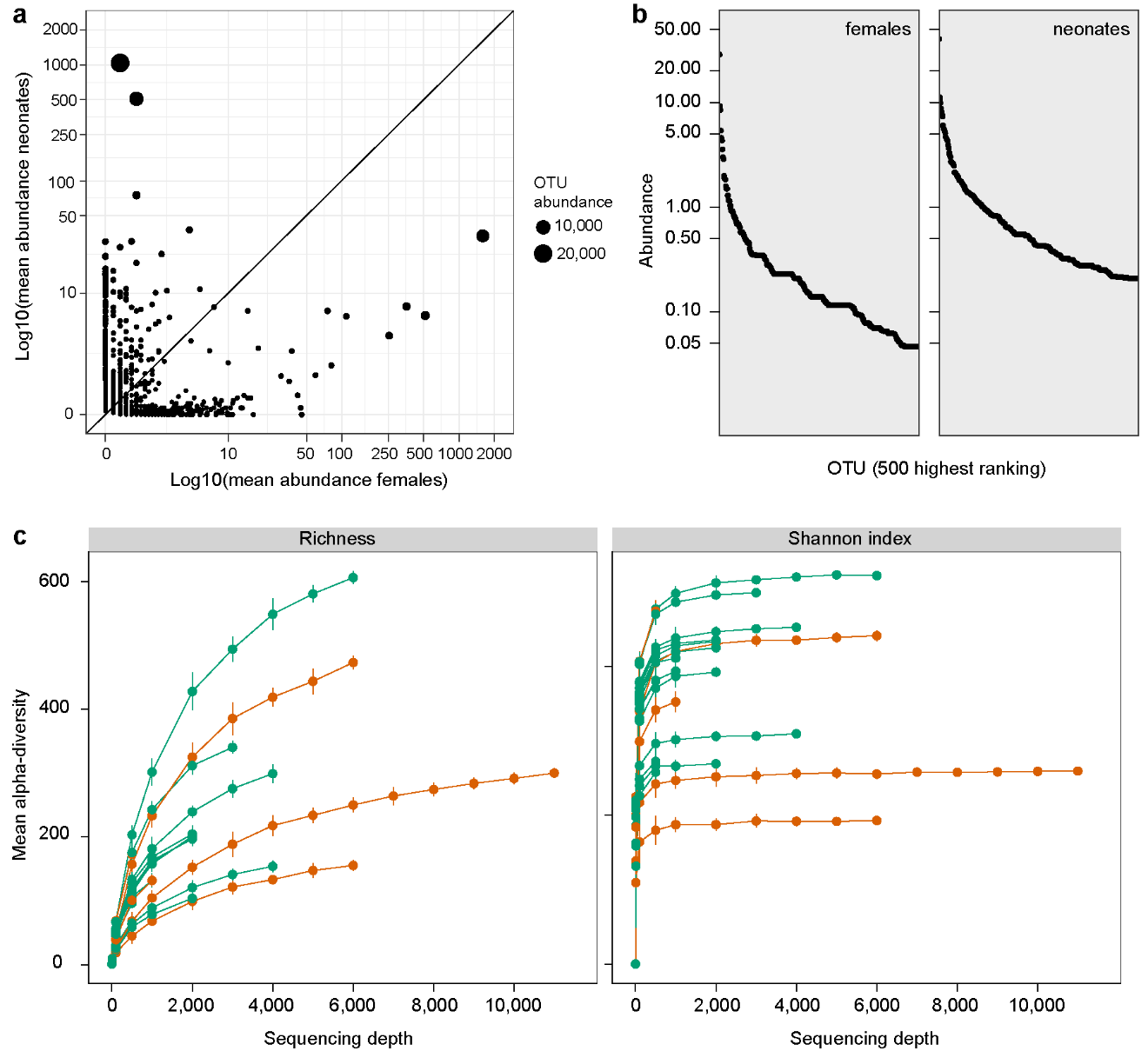


**Fig. S2.** Mean abundance per OTU, rank abundance plots and rarefaction curves. (**a**) Mean abundance per OTU for neonates versus females. Please note that the three outlier neonate samples are not yet removed from the data, but the singleton OTUs are. The two outlier *Staphylococcus* OTUs can be found in the top left part of the graph. The black line indicates *Y*=*X*. Symbol size indicates overall OTU abundance summed over all samples (neonates + females). Note the log-scales. (**b**) Rank abundance plots of the 500 most abundant OTUs for females and neonates (neonatal outlier samples removed). Note the log10 transformation of the Y-axis. Females and neonates show a skewed pattern, with few very abundant OTUs and many rare ones, albeit more clearly so in females. (**c**) Rarefaction curves of female and neonate samples, depicting mean OTU richness and Shannon diversity (error bars represent 2**SD*). Although OTU richness did not reach saturation, the Shannon diversity did level off around 1000 read counts. Green symbols are neonates, orange symbols are females.


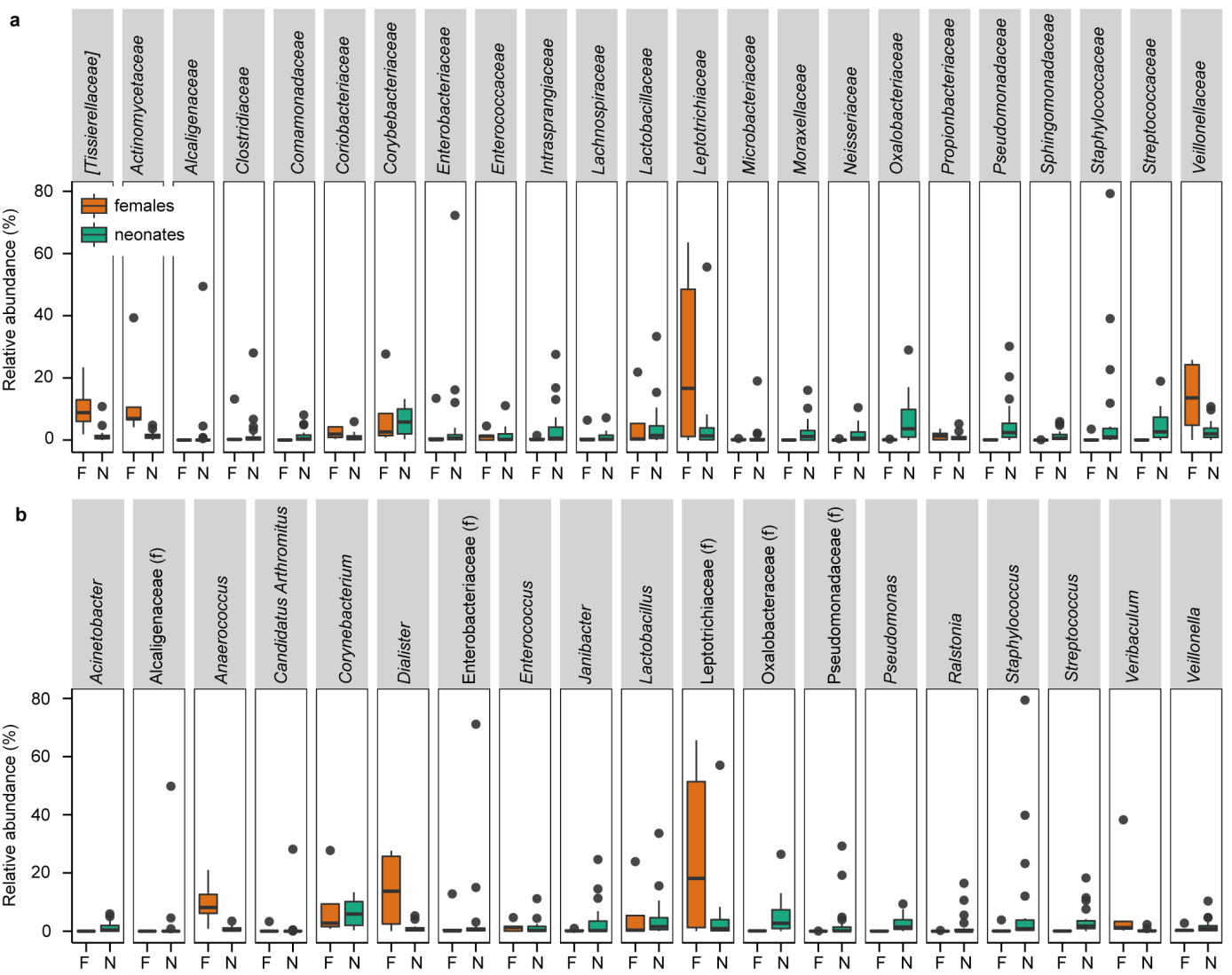
**Fig. S3.** The relative abundances of the most common families (**a**) and genera (**b**) (i.e., relative abundance >1%). Females (orange) and neonates (green) shared many taxa. Boxplots present the median, 25th and 75th percentiles and, if applicable, outliers (black symbols).


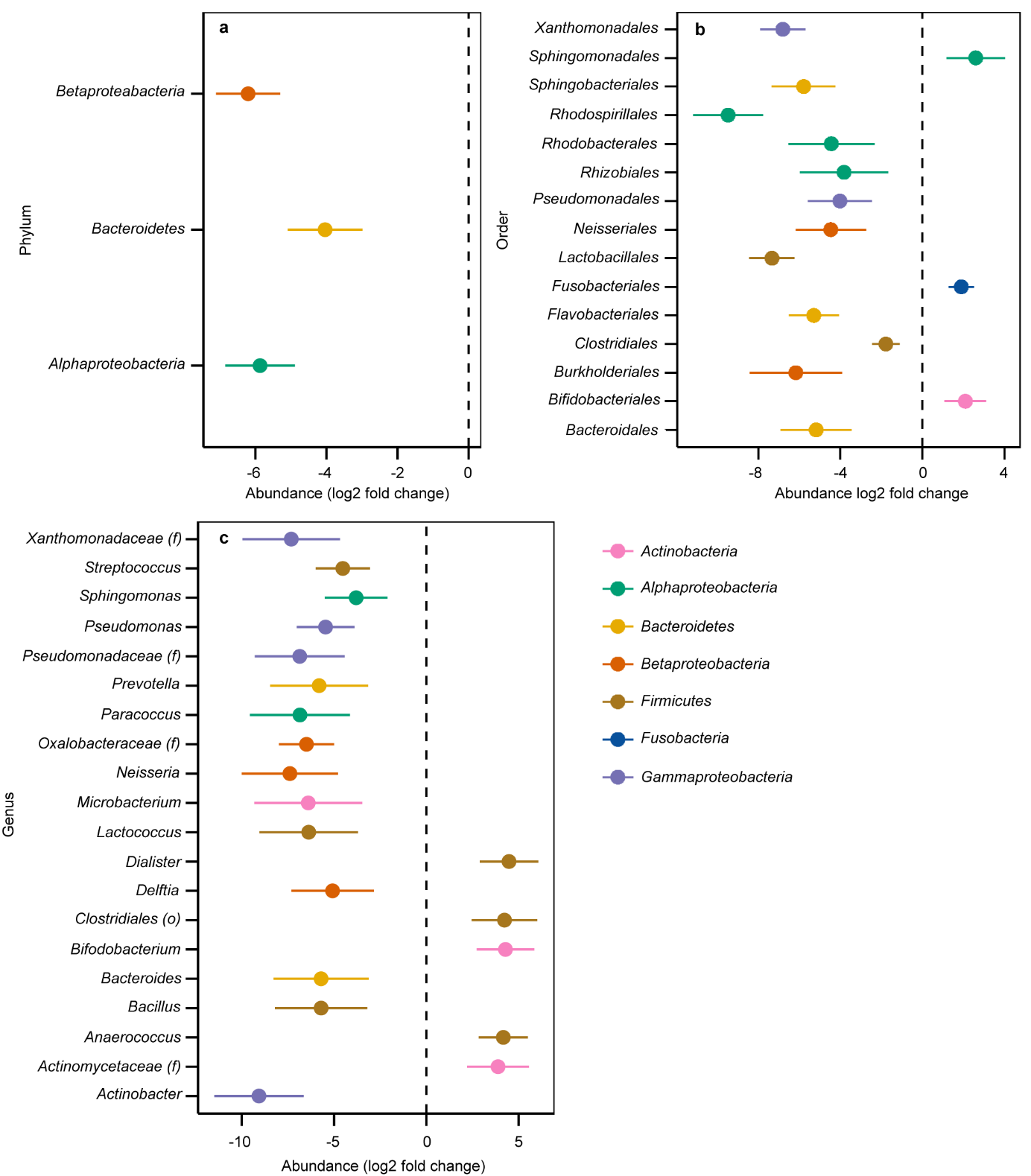


**Fig. S4.** Significant differences in *DESeq2* transformed abundances (FDR *q*<0.1) between neonates and females at “phylum” (**a**), order (**b**), and genus level (**c**). Positive log2 fold changes indicate increased abundances in adult females, whereas negative log2 fold changes indicate increased abundances in neonates. Depicted are means with 95% confidence intervals. Symbol colours refer to “phylum”.


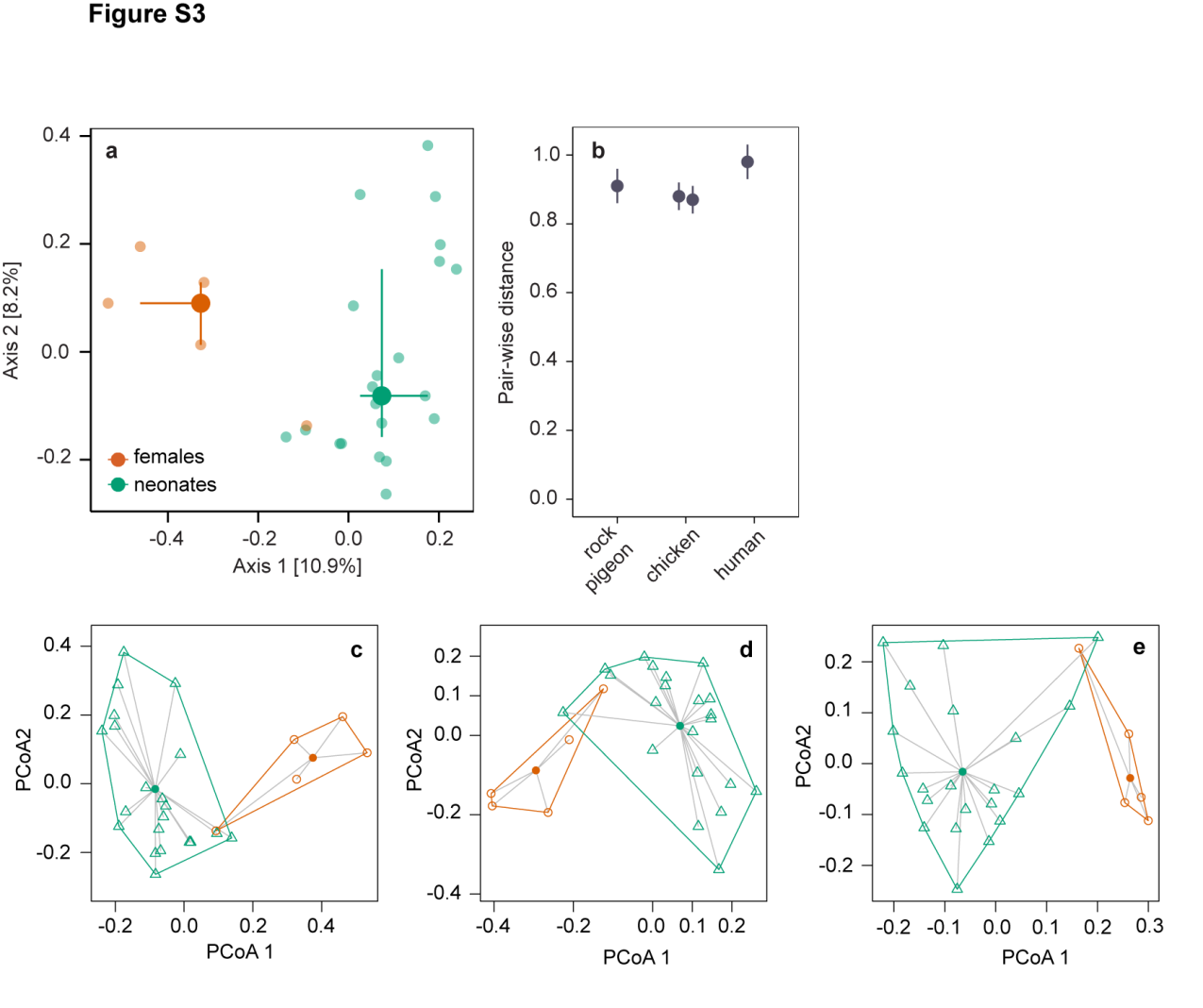


**Fig. S5.** Bray-Curtis distances of rock pigeon and literature data, and group dispersion plots of all beta-diversities. (**a**) PCoA plot of Bray-Curtis distances of rock pigeon neonates (green symbols) and females (orange symbols). Large symbols present the medians, the errors bars the 25% and 75% quantiles. Transparent symbols present the underlying data. (**b**) Mean pair-wise Bray-Curtis distances (± SD) between rock pigeon neonates and females (*n*=105), between chicken females and embryos of 4 (*n*=324) and 19 days (*n*=288) of incubation (19), and human females and neonates (*n=*7488 (26). Group dispersions of (**c**) Bray-Curtis dissimilarities, (**d**) Unweighted Unifrac, and (**e**) weighted UniFrac distances indicate that differences found in beta-diversities are not due to differences in group dispersion.

**Table S1.** Overview of the 311 OTUs present in the five negative control samples.

| **Phylum** | **Class** | **Order** | **Family** | **Genus** | **Species** |
| --- | --- | --- | --- | --- | --- |
| *Actinobacteria* | *Actinobacteria*  *Coriobacteriia*  *Thermoleophilia* | *Actinomycetales*  *Bifidobacteriales*  *Coriobacteriales*  *Solirubacterales* | *Actinomycetaceae*  *Cellulomonadaceae*  *Corynebacteriaceae*  *Dermabacteraceae*  *Dermacoccaceae*  *Frankiaceae*  *Intrasporangiaceae*  *Microbacteriaceae*  *Micrococcaceae*  *Mycobacteriaceae*  *Nordicardiaceae*  *Propionibacteriaceae*  *Bifidobacteriaceae*  *Coriobacteriaceae*  *Conexibacteraceae*  *Patulibacteraceae*  *1x UA* | *Actinomyces*  *mobiluncus*  *Varibaculum*  *Cellulomonas*  *Corynebacterium*  *Brachybacterium*  3x UA  *Dermacoccus*  *Frankia*  *Janibacter*  *Microbacterium*  *Salinibacterium*  7x UA  1x UA  *Mycobacterium*  *Rhodococcus*  *Proprionibacterium*  *Bifidobacterium*  *Scardovia*  4x UA  3x UA  1x UA  1x UA | 8x UA  4x UA  3x UA  1xUA  33x UA  *conglomeratum*  1x UA  1x Ua  5x UA  2x UA  4x UA  *celatum*  1x UA  *fascians*  1x UA  *acnes (3)*  1x UA  1x UA |
| *Bacteroidetes* | *Bacteroidia*  *Flavobacteriia*  *Sphingobacteriia* | *Bacteroidales*  *Flavobacteriales*  *Sphingobacteriales* | *Bacterdoidacaea*  1x UA  *[Weeksellaceae]*  1x UA | *Bacteroides*  *Chryseobacterium* | 2x UA  1x UA |
| *Chloroflexi* | *SAR202* | 1x UA |  |  |  |
| *Cyanobacteria* | *Oscillatoriophycideae* | *Chroococcales* | *Xenococcaceae* | 1x UA |  |
| *Firmicutes* | *Bacilli*  *Clostridia*  *Erysipelotrichi* | *Bacillales*  *Lactobacillales*  *Turicibacterales*  *Clostridiales*  *Erysipelotrichales* | *Staphylococcaceae*  *Enterococcaceae*  *Lactobacillaceae*  *Streptococcaceae*  *Turicibacteraceae*  *[Tissierellaceae]*  *Christensenellaceae*  *Clostridiaceae*  *Lachnospiraceae*  *Peptococcaceae*  *Ruminicoccaceae*  *Veillonellaceae*  *3x UA*  *Erysipelotrichaceae* | *Staphylococcus*  *Enterococcus*  *Enterococcus*  *Lactobacillus*  *Lactobacillus*  *Lactobacillus*  *Streptococcus*  1x UA  *Turicibacter*  *Peptoniphilus*  *ph2*  *Tissierella_Soehngenia*  1x UA  1x UA  *Candidatus Arth.*  *Clostridium*  5x UA  *Coprococcus*  2x UA  *Peptococcus*  2x UA  *Veillonella*  *Veollonella*  *[Eubacterium]* | 4x UA  *cecorum (5)*  4x UA  *agilis (6)*  *ruminis*  25x UA  3x UA  1x UA  1x UA  1x UA  1x UA  7x UA  2x UA  1x UA  1x Ua  *dispar*  2x UA  *cylindroides* |
| *Fusobacteria* | *Fusobacteriia* | *Fusobacteriales* | *Fusobacteriaceae*  *Leptotrichicaceae* | *Fusobacterium*  *Leptotrichia* | *1x UA*  *1x UA* |
| *Proteobacteria* | *Alphaproteobacteria*  *Betaproteobacteria*  *Gammaproteobacteria* | *Caulobacterales*  *Rhizobiales*  *Rhodobacterales*  *Sphingomodales*  *Burkholderiales*  *Enterobacteriales*  *Legionellales*  *Pasteurellales*  *Pseudomodales*  *Xanthomodales* | *Caulobacteraceae*  *Aurantimodaceae*  *Methylobacteriaceae*  *Methylocystaceae*  *Rhodobacteraceae*  *Sphingomonadaceae*  *Alcaligeceae*  *Alcaligaceae*  *Alcaligaceae*  *Comamodaceae*  *Enterobacteriaceae*  *Coxiellaceae*  *Pasteurellaceae*  *Moraxellaceae*  *Pseudomodaceae*  *Xanthomodaceae* | 1x UA  1x UA  *Methylobacterium*  2x UA  *Paracoccus*  *Sphingomos*  *Sphingomos*  *Achromobacter*  *Sutterella*  5x UA  *Comamos*  *Delftia*  13x UA  10x UA  *Escherichia*  *Plesiomos*  *Proteus*  32x UA  *Rickettsiella*  *Gallibacterium*  *Acinetobacter*  *Pseudomos*  *Pseudomos*  *Luteimos* | 1x UA  2x UA  *azotifigens*  *yabuuchiae (8)*  1x UA  3x UA  1x UA  4x UA  *coli*  *shigelloides*  1x UA  4x UA  2x UA  2x UA  *viridiflava*  7x UA  1x UA |
| *Tenericutes* | *CK-1C4-19*  *Mollicutes* | 1x UA  1x UA |  |  |  |
| *WPS-2* | *1x UA* |  |  |  |  |
| *Unassigned (190x)* |  |  |  |  |  |

If more than 1 OTU was present per bacterial strain, this is indicated with a number between brackets. OTUs not assigned to taxonomic level are indicated with UA, including the number of OTUs at that level. 190 OTUs were unassigned at kingdom level.

**Table S2.** Overview of phyla, orders, genera and OTUs differing in normalized (*DESeq2*) or relative abundance.

| Taxa level | Differences in normalized abundance in: | | Differences in relative abundance in: |
| --- | --- | --- | --- |
| “Phylum” | *Alphaproteobacteria*  *Bacteroidetes*  *Betaproteobacteria* | *Actinobacteria Betaproteobacteria* | *Actinobacteria*  *Betaproteobacteria* |
| Order | *Bacteroidales*  *Bifidobacteriales*  *Burkholderiales*  *Clostridiales*  *Flavobacteriales*  *Fusobacteriales*  *Lactobacillales*  *Neisseriales* | *Actinomycetales*  *Bacteroidales*  *Bifidobacteriales*  *Burkholderiales*  *Clostridiales*  *Pseudomonadales* | *Pseudomonadales*  *Rhizobiales*  *Rhodobacterales*  *Rhodospirillales*  *Sphingobacteriales*  *Sphingomonadales*  *Xanthomonadales* |
| Genus | *Acinetobacter*  *Actinomycetaceae (f)*  *Anaerococcus*  *Bacillus*  *Bacteroides*  *Bifidobacterium*  *Clostridiales (o)*  *Delftia*  *Dialister*  *Lactococcus* | *Anaerococcus*  *ph2*  *Varibaculum* | *Microbacterium*  *Neisseria*  *Oxalobacteraceae (f)*  *Paracoccus*  *Prevotella*  *Pseudomonadaceae (f)*  *Pseudomonas*  *Sphingomonas*  *Streptococcus*  *Xanthomonadaceae (f)* |
| OTU | NA |  | *Actinomycetaceae (f; 2x)*  *Anaerococcus (g)*  *Atopobium (g; 2x)*  *Bifidobacterium (g)*  *Clostridiales (o; 2x)*  *Corynebacterium (g)*  *Micrococcaceae (f)*  *Streptococcus (g)*  Unassigned (4x) |

When OTUs were defined at higher taxonomic levels, this is indicated between brackets (o = order, f = family, g = genus). When multiple OTUs within a level differed significantly between neonates and females, this is indicated by a number between brackets. None of the OTUs differed in absolute abundance between neonates and females. Normalized abundances differed significantly at FDR<0.1 (*DESeq2*), relative abundances differed significantly at FDR<0.05 (*ANCOM*).

**Table S3.** Overview of the OTUs present in core microbiome of neonates and females.

| **Phylum** | **Class** | **Order** | **Family** | **Genus** | **Species** |
| --- | --- | --- | --- | --- | --- |
| ***Neonates***  *Actinobacteria* | *Actinobacteria* | *Actinomycetales* | *Corynebacteriaceae* | *Corynebacterium* | 1x UA |
| *Firmicutes* | *Bacilli*  *Clostridia* | *Bacillales*  *Lactobacillales*  *Clostridiales* | *Staphylococcaceae*  *Streptococcaceae*  *[Tissierellaceae]*  *Peptococcaceae*  *Veillonellaceae* | *Staphylococcus*  *Streptococcus*  *Anaerococcus*  *Peptococcus*  *Dialister*  *Veillonella* | *2x UA*  *3x UA*  *1x UA*  *1x UA*  *2x UA*  *dispar* |
| *Fusobacteria* | *Fusobacteriia* | *Fusobacteriales* | *Leptotrichiaceae* | 1x UA |  |
| ***Females***  *Actinobacteria* | *Actinobacteria*  *Coriobacteriia* | *Actinomycetales*  *Bifidobacteriales*  *Coriobacteriales* | *Actinomycetaceae*  *Corynebacteriaceae*  *Micrococcaceae*  *Propionibacteriaceae*  *Bifidobacteriaceae*  *Cirobacteriaceae* | *Varibaculum*  3x UA  *Corynebacterium*  1x UA  1x UA  *Bifidobacterium*  *Atopobium* | 1x UA  4x UA  1x UA  3x UA |
| *Firmicutes* | *Clostridia* | *Clostridiales* | *[Tissierellaceae]*  *Veillonellaceae*  1x UA | *Aerococcus*  *Peptoniphilus*  *ph2*  *Dialister*  *Veillonella* | 1x UA  2x UA  1x UA  2x UA  *dispar* |
| *Fusobacteria* | *Fusobacteriia* | *Fusobacteriales* | *Leptotrichicaceae* | 2x UA |  |
| UA (4x) |  |  |  |  |  |

OTUs were include in the core microbiome if present in 60% of the samples. OTUs not assigned to a taxonomic level are indicated with UA, including the number of OTUs at that level. Four OTUs were unassigned at kingdom level.
